# RADseq provides evidence for parallel ecotypic divergence in the autotetraploid *Cochlearia officinalis* in Northern Norway

**DOI:** 10.1038/s41598-017-05794-z

**Published:** 2017-07-17

**Authors:** Marie K. Brandrud, Ovidiu Paun, Maria T. Lorenzo, Inger Nordal, Anne K. Brysting

**Affiliations:** 1 Centre for Ecological and Evolutionary Synthesis, Department of Biosciences, University of Oslo, 0316 Oslo, Norway; 2 Department of Botany and Biodiversity Research, University of Vienna, 1030 Vienna, Austria; 3 Department of Biosciences, University of Oslo, 0316 Oslo, Norway

## Abstract

Speciation encompasses a continuum over time from freely interbreeding populations to reproductively isolated species. Along this process, ecotypes – the result of local adaptation – may be on the road to new species. We investigated whether three autotetraploid *Cochlearia officinalis* ecotypes, adapted to different habitats (beach, estuary, spring), are genetically differentiated and result from parallel ecotypic divergence in two distinct geographical regions. We obtained genetic data from thousands of single nucleotide polymorphisms (SNPs) from restriction-site associated DNA sequencing (RADseq) and from six microsatellite markers for 12 populations to assess genetic divergence at ecotypic, geographic and population level. The genetic patterns support differentiation among ecotypes as suggested by morphology and ecology. The data fit a scenario where the ancestral beach ecotype has recurrently and polytopically given rise to the estuary and spring ecotypes. Several ecologically-relevant loci with consistent non-random segregating patterns are identified across the recurrent origins, in particular around genes related to salt stress. Despite being ecologically distinct, the *Cochlearia* ecotypes still represent an early stage in the process of speciation, as reproductive isolation has not (yet) developed. A sequenced annotated genome is needed to specifically target candidate genes underlying local adaptation.

## Introduction

Speciation often occurs as a continuous process over time from freely interbreeding populations to reproductively isolated species^1–3^. Along this continuum, ecotypes may be formed as a result of local adaptation to specific sets of environmental factors that define different habitats^4^. Even though the ecotype concept and its role in plant speciation have been subject to heavy debate during the last century^2^, several empirical studies show non-random organisation of morphological and genetic variation related to more or less steep ecological gradients^4–6^, supporting that ecotypes could be considered non-static entities along the speciation continuum. The study of adaptive divergence between ecotypes may, thus, be an important contribution for understanding the process of speciation. Instances of parallel ecotypic divergence where adaptation to similar conditions repeatedly cause similar phenotypic changes in closely related organisms are especially useful for disentangling the respective roles of drift and natural selection in shaping genomic divergence among genomes and for studying the genes underlying local adaptation^7^. The ecological variation found among populations of the autotetraploid *Cochlearia officinalis* in northern Norway potentially represents a highly valuable system to explore parallel ecotypic differentiation in plants.

The genus *Cochlearia* (Brassicaceae) constitutes a good example of a group of recently evolved, and in some cases not yet fully differentiated taxa, which most likely diversified during the mid or late Pleistocene^8–10^. The taxa inhabit coastal and inland (alpine) habitats and are distributed throughout Central and Northern Europe, extending the distribution of the genus into the arctic region^11^. Most taxa are dependent on a good supply of water or moist soil conditions throughout the year, and parallel adaptation to different types of moist habitats may be important for the diversification within the group^12, 13^. The taxa together exhibit complex variation not only with regard to ecology and morphology, but constitute also a polyploid complex of diploids, tetraploids, hexaploids, and octoploids^9, 10, 14–18^.

**Figure 1:**
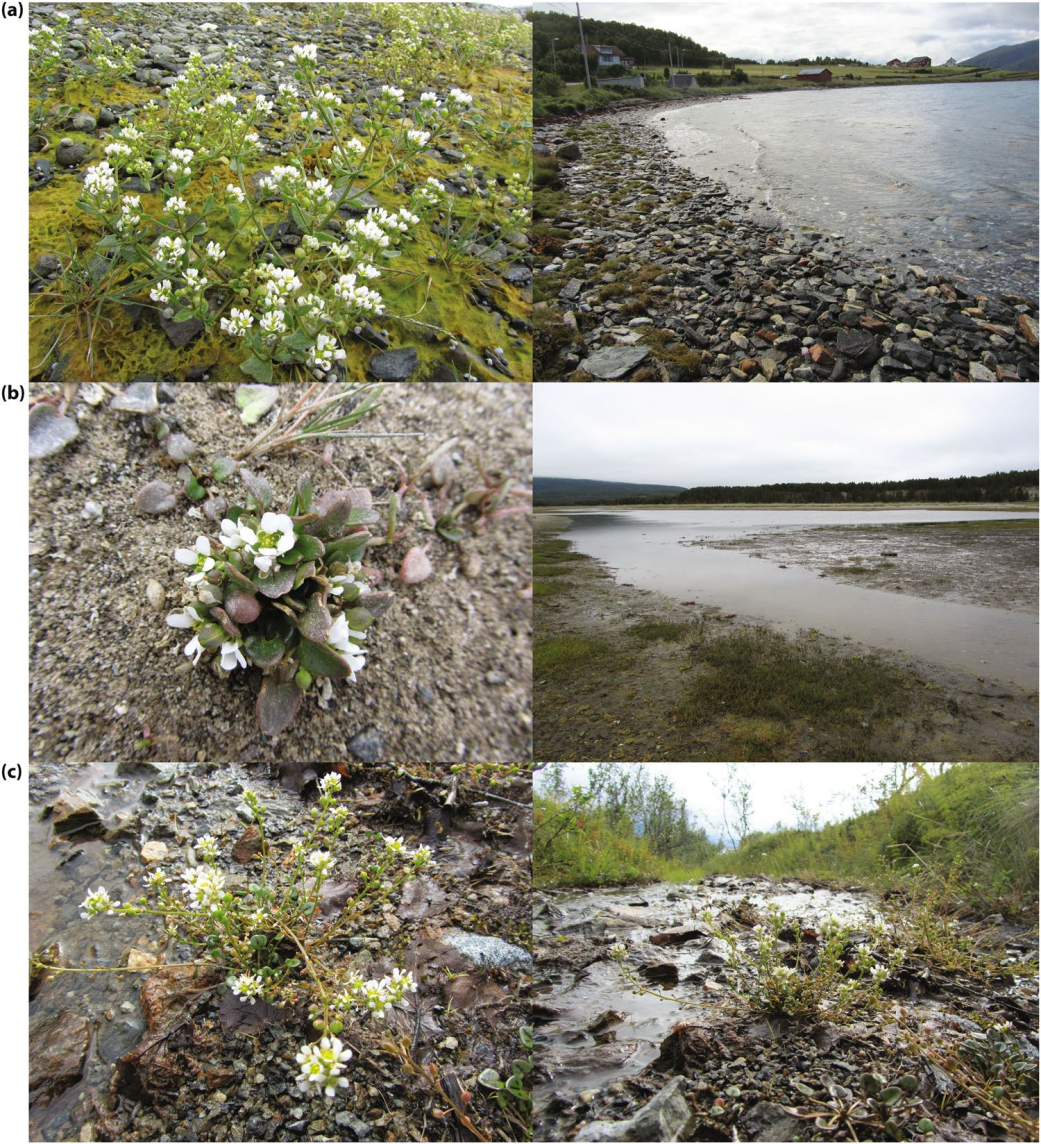
Habit and habitat of the three ecotypes of *Cochlearia officinalis* (2*n = *24) in Troms, Norway. (**a**) The beach ecotype (ssp. *officinalis*) at localities Sjøvassbotn (left) and Skittenelv (right), (**b**) the estuary ecotype (ssp. *norvegica*) at locality Skibotn, and (**c**) the spring ecotype (ssp. *integrifolia*) at locality Kvaløysletta. (Photo: M.K. Brandrud).

The tetraploid *C*. *officinalis* is a cold-tolerant halophyte, widely distributed along the European coastline^14, 19, 20^. Gill suggested based on studies of chromosome associations during meiosis in F1 hybrids, that *C*. *officinalis* originated by autopolyploidy from the Central European *C*. *pyrenaica* ^17^. Molecular data support an autotetraploid origin, although not directly from present day diploids^10^. Previous studies have found morphological and ecological variation in Northern Scandinavia, which has been suggested to represent differentiation at the ecotypic level, and several subspecies have been recognised^16, 20, 21^. The common beach ecotype, or ssp. *officinalis*, grows in gravel beaches (Fig. 1a), crevices in beach cliffs (Supporting Information Fig. S1a), salt marshes and occasionally in bird cliffs, where it shows vigorous growth and seems to be adapted to exploit the high nutrient levels^13, 16^. The estuary ecotype, or ssp. *norvegica*, grows in sheltered habitats near outlets of large rivers in innermost fjords (Fig. 1b, Supporting Information Fig. S1b), which are inundated by brackish water at flood-tide^13, 16^. This ecotype seems to be adapted to handle nutrient poor habitats and shows very little increase in growth when presented with higher nitrogen levels^21^. The spring ecotype, or ssp. *integrifolia*, grows inland in more or less base-rich cold springs (Fig. 1c, Supporting Information Fig. S1c), along streams and brooks or in snow beds^13, 16^.

Nordal and Stabbetorp found that the three ecotypes are not only ecologically differentiated but also to some degree morphologically distinct^16^. However, no single quantitative character unambiguously separates the three ecotypes^16^. The morphologically most distinct ecotype is the estuary ecotype, with larger flowers and cuneate (as opposed to more or less kidney-shaped) rosette leaves that are fleshier than those of the other two ecotypes. In the beach ecotype, the fruit (silicula) is more spherical in outline than in the estuary and spring ecotypes. The spring ecotype has a tendency to be perennial rather than biennial as is the case for the two other ecotypes. This is indicated by a branching rhizome that gives rise to more rosettes, and by the development of buds before the snow has melted. When comparing plants collected in the field with plants cultivated in common conditions, distinctiveness in flower and fruit characters tended to be stable, whereas the size and shape of rosette and stem leaves, as well as the branching and elongation of inflorescences, were highly plastic depending on environmental conditions^16^.

Genetic studies of *Cochlearia* so far^10, 14, 22–25^ have not included plants representing the ecotypic variation found in Northern Scandinavia. Using several thousand single nucleotide polymorphisms (SNPs) obtained from restriction-site associated DNA sequencing (RADseq), and microsatellite markers, we explore here whether and to what degree the ecotypes are genetically differentiated. We infer the genetic structure of the three ecotypes from two geographical areas of Northern Norway and we ask whether the ecological and morphological variation that we see today is the result of parallel evolution through local adaptation to different habitats.

## Results

### RADseq analyses

**Figure 2:**
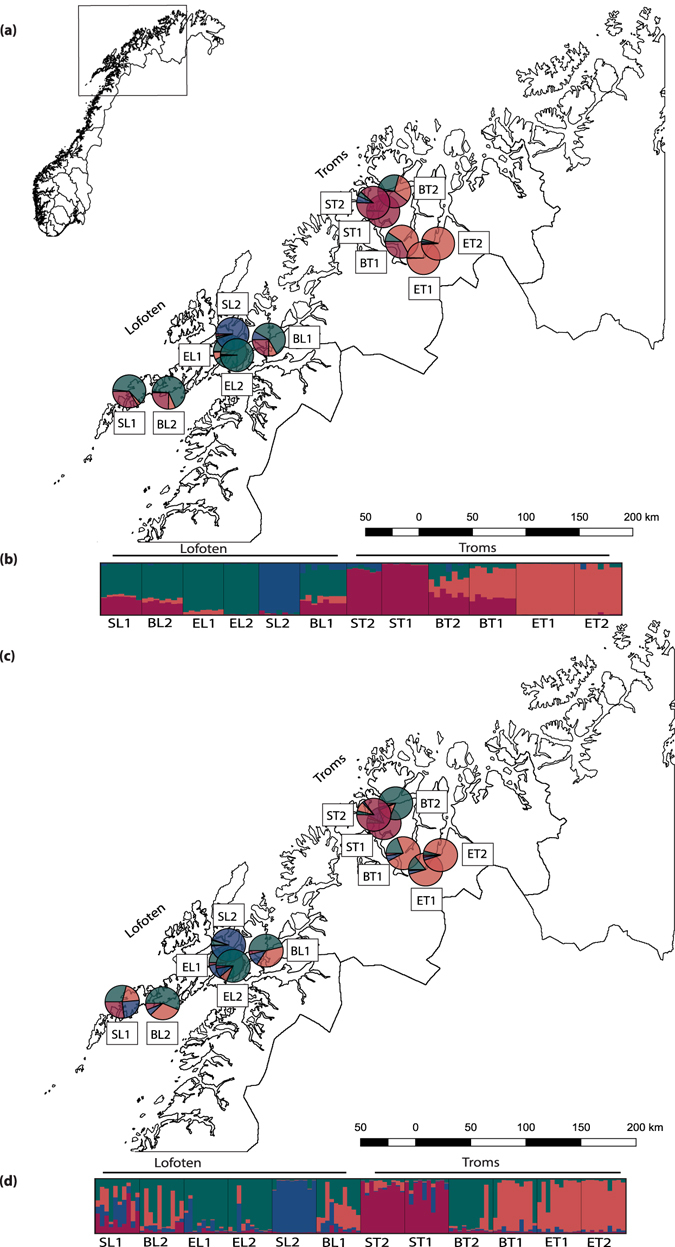
Genetic structure of 12 populations of *Cochlearia officinalis* in Northern Norway. (**a**,**b**) Results from STRUCTURE analysis (K = 4) performed on 89 individuals, using 4,296 SNPs from the RADseq data. (**c**,**d**) Results from STRUCTURE analysis (K = 4) performed on 120 individuals, using microsatellite allele sizes. (**a**,**c**) Overall assignment of populations to the four STRUCTURE groups visualized by pie charts on a map of Northern Norway. The two sampling areas are indicated as Troms and Lofoten. (**b**,**d**) Proportional assignment of individuals to the four STRUCTURE groups. Each individual is represented by a bar and populations are separated by a black line. Populations are named according to ecotype (B = beach, E = estuary, S = spring) and geography (T = Troms, L = Lofoten), see Table 1. The map layer was extracted from GADM version 1.0^72^.

Plant material of the three *C*. *officinalis* ecotypes (beach, estuary and spring) were sampled in Northern Norway in two areas (Fig. 2, Table 1) where they broadly co-occur: Tromsø-Skibotn in Troms county (in the following called Troms) and Lofoten in Nordland county (in the following called Lofoten). Fifty-four individuals from 12 populations, representing the three ecotypes (Table 1), were analysed by flow cytometry to estimate ploidal level, and all were confirmed to be tetraploid.

**Table 1:** Collection data for *Cochlearia officinalis* L. populations from Northern Norway.

Ecotype (taxon)	Pop. ID	Locality	Latitude/longitude (degrees)	No. of individuals analysed			Collection date (voucher no.)
				RADseq	Micro-satellites	Flow cytometry	
**Spring** (ssp. *integrifolia* (Hartm.) Nordal & Stabbetorp)	SL1	Nordland, Vestvågøy, Himmeltind (70–345 m a.s.l.)	68.21663/13.52911–13.54613	8	10	2	12.08.2013 (MKB13-4)
	SL2	Nordland, Sortland, Sørfjorddalen (80 m a.s.l.)	68.67430/15.77752	8	10	2	13.08.2013 (MKB13-7)
	ST1	Troms, Tromsø, Tromsdalen (40 m a.s.l.)	69.62466/19.04558	6	10	3	18.08.2012 (MKB12-6)
	ST2	Troms, Tromsø, Kvaløysletta (65–70 m a.s.l.)	69.68858/18.83241	7	10	5	18.08.2012 (MKB12-7)
**Estuary** (ssp. *norvegica* Nordal & Stabbetorp)	EL1	Troms, Kvæfjord, Gullesfjordbotnen	68.53358/15.72755	7	10	2	13.08.2013 (MKB13-6)
	EL2	Nordland, Lødingen, Kanstadbotnen	68.50711/15.87602	10	10	2	14.08.2013 (MKB13-8)
	ET1	Troms, Storfjord, Melneset, Oteren	69.26708/19.92058	7	10	9	16.08.2012 (MKB12-2)
	ET2	Troms, Storfjord, Skibotn	69.37866/20.23541	7	10	10	16.08.2012 (MKB12-4)
**Beach** (ssp. *officinalis*)	BL1	Troms, Harstad, Tjeldsundbrua, Tjeldsundet	68.62775–68.62966/16.56533–16.56900	6	10	2	MKB13-3 (11.08.2013)
	BL2	Nordland, Vågan, Ørsnes	68.20463/14.39763	8	10	2	MKB13-9 (14.08.2013)
	BT1	Troms, Tromsø, Sjøvassbotn, Sørfjord	69.39397/19.45308	7	10	7	MKB12-1 (16.08.2012)
	BT2	Troms, Tromsø, Skittenelv	69.77505/19.29294	8	10	8	MKB12-8 (18.08.2012)

Pop ID: Population ID according to ecotype (B = beach, E = estuary, S = spring) and geography (T = Troms, L = Lofoten). When m a.s.l. is not given, the population was sampled at sea level. Number of individuals used for RADseq, microsatellite analyses and flow cytometry is given. Collectors: M.K. Brandrud, I. Nordal, A.K. Brysting.

From c. 428 million raw paired-end reads obtained from RADseq, c. 163 million forward reads were retained after demultiplexing and cleaning. The second reads in the pairs were only used in the process of demultiplexing based on combinatorial inline barcodes and for extending contigs for the annotation of outlier loci. After *de novo* catalog building and SNP calling, we retained c. 15.000 high-quality loci present in at least 80% of the 89 individuals included in the analysis (Table 1). These were further filtered with various parameters to construct input files for population genetic and phylogenetic analyses (Supporting Information Table S1). Given that the 1C genome size of *C*. *officinalis* is estimated to 0.75 pg^26^, i.e. 734 megabases (Mbp), and following the procedure in Lowry *et al*.^27^, the retained RAD loci density in the current study was estimated to be 5,329 RAD loci over 734 Mbp, i.e. 7.26 RAD loci/Mbp.

The number of private alleles were highest in the beach ecotype (661) compared to the estuary (603) and spring (494) ecotypes (Supporting Information Table S2). All populations had negative inbreeding coefficients (F_IS_) when calculated from the RADseq data, indicating an excess of heterozygotes, estimates confirmed also with our microsatellite analyses (see below). It should be noted that when analysing RADseq data in STACKS, F_IS_ is calculated as 1-(H_o_/H_e_). For polyploids we would, however, expect higher levels of heterozygosity than for diploids^28^, meaning that in our calculations F_IS_ is most likely underestimated by using this approach. The spring population from Sørfjorddalen in Lofoten (SL2) had a lower number of private alleles, and an inbreeding coefficient closer to zero than other populations. This population grows in a spring in an open forest area relatively far from the sea (Fig. S1c). The second spring population from Lofoten (Himmeltind, SL1), which grows in a small stream near the outlet to the sea, had a higher number of private alleles and a more negative inbreeding coefficient, comparable to what we found for the beach ecotype in the same area (Supporting Information Table S2).

The number of migrants based on private alleles (Nm) was subunitary in all population pairs, suggesting an important role for drift (and/or local selection) in shaping the genetic structure of the group. In general, the beach populations had highest connectivity, independent of geographic distance. The other ecotypes were less connected by gene flow (except for the estuary populations from Troms), with the number of migrants within ecotypes not different from that between ecotypes or between regions. The lowest levels of gene flow were found for the isolated spring population from Lofoten (SL2), followed by the two spring populations from Troms. Highest number of migrants was found between the two estuary populations from Troms, which are both geographically and genetically close, followed by the beach populations from both areas (Supporting Information Fig. S2, Table S3).

Analyses of molecular variance (AMOVA; Supporting Information Table S4) showed that most of the variation in the dataset was found within populations (with heterozygosity as an important part of the total variation, but see above comments on F_IS_ estimates). Although only a small percentage (c. 5%) was explained by differences between ecotypes, this part was larger than the variation explained by the two geographic regions (2%).

**Figure 3:**
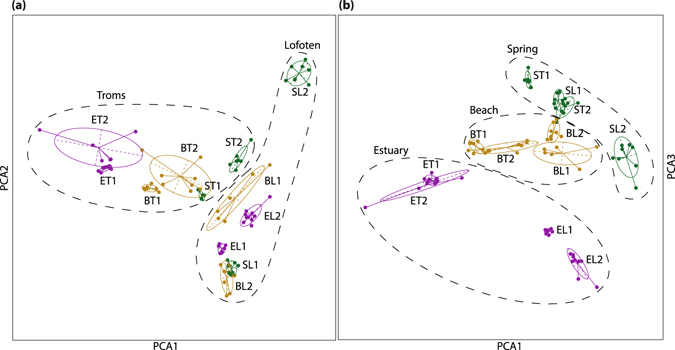
PCA performed on 12 populations of *Cochlearia officinalis*, using 4,296 SNPs from the RADseq data. (**a**) The first and second PCA axes. (**b**) The first and third PCA axes. The first PCA axis explains 4.05%, the second axis 3.82%, and the third axis 3.46% of the total variation. Colour represents ecotypes: estuary - purple, beach - yellow and spring - green. Individuals (dots) are linked to the centre of the 95% inertia ellipse for the population to which they belong. Populations are named according to ecotype (B = beach, E = estuary, S = spring) and geography (T = Troms, L = Lofoten), see Table 1.

In a principal component analysis (PCA), based on 4,296 SNPs, the first three axes explained 11.3% of the variation in the data (Fig. 3). Taken together, the three axes separated all populations except for some overlap between the two estuary populations from Troms and between the two beach populations from Troms. Further, the main signal followed the ecotypic differentiation (the first and third axes in combination) in addition to a (weaker) geographical separation between Troms and Lofoten (first and second axis in combination). Overall, the beach populations were less well separated and localised at the centre of the plot. The isolated spring population from Lofoten (SL2) was the most distinct population, whereas the more exposed spring population from Lofoten (SL1) overlapped with one of the beach populations from the same area. Although more vaguely, the same tendency was seen in Troms; the isolated forest spring population (Tromsdalen, ST1) was genetically more distinct from the beach populations than the spring population growing in a somewhat more exposed area (Kvaløysletta, ST2; Fig. 1c).

From a STRUCTURE analysis, based on 4,296 SNPs, K = 4 was selected based on the optimal deltaK and the mean likelihood value (followed by K = 2 and K = 9, Supporting Information Fig. S3). When the STRUCTURE results were plotted on a map of Northern Norway as pie charts representing the affiliation of single populations to the four STRUCTURE groups (Fig. 2a), a geographical pattern was seen with two of the groups (‘purple’ and ‘orange’) dominating in Troms, and the other two groups (‘blue’ and ‘green’) dominating in Lofoten. Overall, the beach populations were genetically similar and showed admixture of three of the four STRUCTURE groups, though with populations in Lofoten and Troms differing in relative allocation to each genetic pool (Fig. 2b). The estuary populations in Lofoten allocated to a STRUCTURE group dominating among the beach populations in Lofoten (‘green’), whereas the estuary populations in Troms allocated to a STRUCTURE group dominating among the beach populations in Troms (‘purple’). The isolated spring population from Lofoten (SL2) was also in this analysis genetically the most distinct of all analysed populations, constituting a genetic group of its own (‘blue’), whereas the second spring population from Lofoten (SL1) was admixed and genetically similar to the beach populations from the same area.

**Figure 4:**
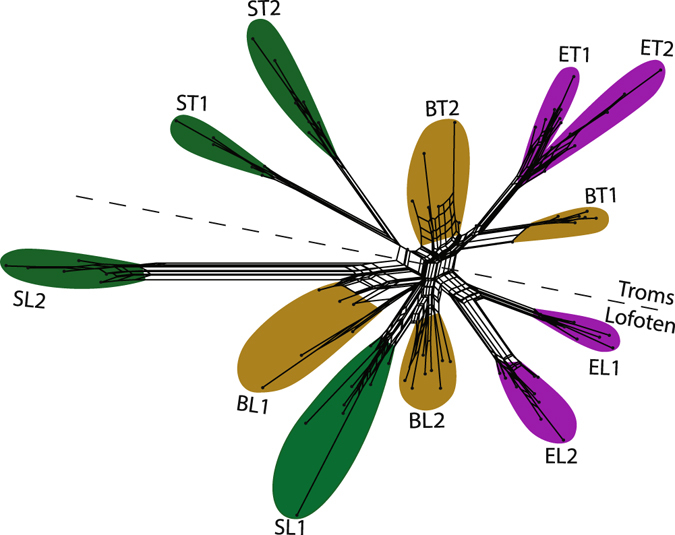
Neighbour net performed on 12 populations of *Cochlearia officinalis*, using 4,311 SNPs from the RADseq data. Colour represents ecotypes: beach - yellow, estuary - purple and spring - green. Populations are named according to ecotype (B = beach, E = estuary, S = spring) and geography (T = Troms, L = Lofoten), see Table 1. The dotted line indicates a geographical split between the two sampling areas, Troms and Lofoten.

In a neighbour net, based on 4,311 SNPs, the individuals clustered according to the 12 populations (Fig. 4). The spring and estuary populations were mainly supported by well-defined splits, whereas the beach populations were less well defined with relatively short splits and a high degree of reticulation corresponding to the high level of admixture seen in the STRUCTURE results. This was also the case for the admixed spring population from Lofoten (SL1). The isolated spring population from Lofoten (SL2) was again the most distinct population, supported by the largest split in the network. There was a relatively clear geographical split across the network, separating populations from the two sampling areas (Troms and Lofoten).

**Figure 5:**
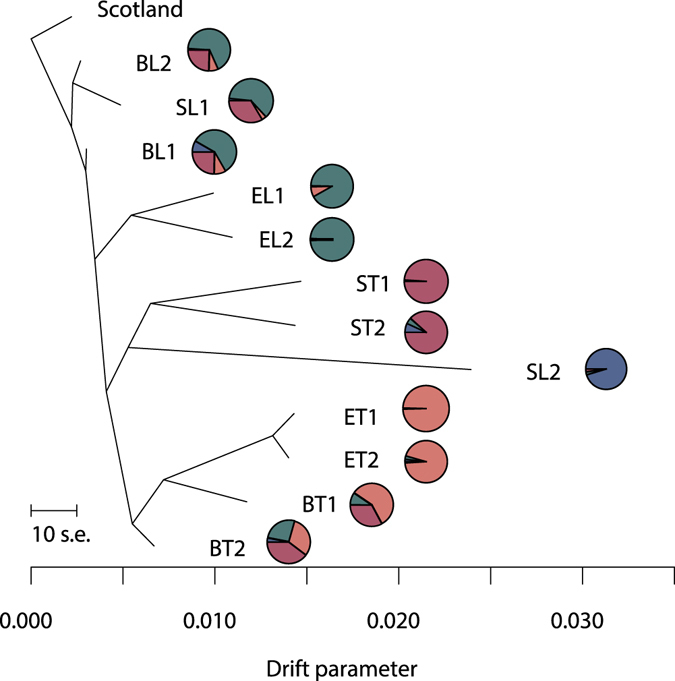
TREEMIX performed on 12 populations of tetraploid *Cochlearia officinalis* from Northern Norway, using 5,982 SNPs from the RADseq data. A population from Scotland (Aberdeenshire) was used as outgroup. Overall assignment of populations to the four STRUCTURE groups is visualized by pie charts (corresponding to Fig. 2a). Populations are named according to ecotype (B = beach, E = estuary, S = spring) and geography (T = Troms, L = Lofoten), see Table 1.

From a TREEMIX analysis, based on 5,982 SNPs and including an outgroup population from Scotland (Aberdeenshire), a tree with no migration events was chosen (Fig. 5; adding individual migration events did not significantly improve the overall residual plot). The tree corresponded well with the STRUCTURE results and further indicated the beach ecotype as the ancestral ecotype, two origins of the estuary ecotype (one in each geographical area), and at least two origins of the spring ecotype. One of these gave rise to the two spring populations in Troms together with the isolated spring population in Lofoten (SL2); the latter, however, on a long branch confirming its distinctiveness. The second Lofoten population (SL1), which grouped with the basal beach populations, may have a separate recent origin. Alternatively, this population may be so heavily influenced from gene flow with the nearby beach populations and isolated from other spring populations for such a long time that it appears genetically closer to the beach ecotype.

A BAYESCAN analysis testing for outlier (RADseq) loci potentially differentiating the beach from the estuary ecotype identified 36 loci within Lofoten and 38 within Troms, with three of the loci in common (Supporting Information Table S5, Fig. S4a). The analysis testing for outlier loci potentially differentiating the beach from the spring ecotype identified 31 loci within Lofoten and 32 within Troms, with four common outliers (Supporting Information Table S5, Fig. S5a). Annotations of the common outliers (Table 2) indicated genes of potential physiological and ecological relevance, e.g. NHX1 and GABA-T that regulate salt and drought stress tolerance. GO enrichment tests for biological processes and molecular functions was obtained for each comparison, but little to no overlap was found between pairwise comparisons in different geographical areas (Supporting Information Figs S4b,c and S5b,c).

**Table 2:** GenBank accession numbers and annotation information for outlier loci from ecotype comparisons.

Locus	GenBank Acc. No.	BLAST annotation	Function in plants
**Beach-Estuary**			
11990	KY580438	Gamma-aminobutyric acid transaminase (GABA-T)	Regulating pH, nitrogen storage, osmolyte activity, development and defence^84^
13110^*^	KY580439	Vacuolar sodium proton antiporter (NHX1)	Regulating salt and drought stress tolerance^44^
18967^*^	KY580440	Solute carrier 35 (nucleoside-sugar transporter)	Transmembrane transport^85^
**Beach-Spring**			
2851	KY580441	Calcium exchanger 7 (CAX7)	Regulating Ca^2+^ homeostasis during abiotic stress and development^86^
2873	KY580442	CRIB domain-containing RIC4-like	Potential molecular switch during development and response to environment^87^
8172	KY580443	Galacturonosyltransferase 10	Synthesis of cell wall^88^
19348^*^	KY580444	AT-hook protein of GA feedback 2 (AGF2)	Gibberellin signaling in development^89^

BLAST annotation is given for outlier loci that were found in both beach-estuary comparisons (i.e. within Troms and within Lofoten) and in both beach-spring comparisons. Outlier loci marked with an asterisk were not specific for a particular ecotype comparison (see Supplementary Information Table S6).

### Microsatellite analyses

Six microsatellites developed for other Brassicaceae taxa (*Arabidopsis*, *Brassica* and *Draba*; Supporting Information Table S6) were successfully co-amplified in *C*. *officinalis* and were further used to analyse the 12 populations from Northern Norway. The six microsatellites each had from two to 39 alleles, and in total 98 alleles were scored for 120 individuals. Even though the F_IS_ values calculated from the microsatellites varied more between populations (Supporting Information Table S2), they were also slightly negative or close to zero (in populations ST1 and EL2 F_IS_ estimated with microsatellites were even lower than RADseq-derived estimates), indicating slight excess of heterozygotes. A STRUCTURE analysis resulted in similar, though less distinct, patterns of genetic variation as obtained from RADseq data. The degree of admixture varied considerably between single individuals within a population. Based on deltaK, K = 3, followed by K = 7 were suggested as representative number of groups (Supporting Information Fig. S6). When K = 4 was selected (for comparison with the four RADseq groups), and population affiliation to STRUCTURE groups was plotted on the map of Northern Norway (Fig. 2c), a similar geographical pattern as for the RADseq data was seen, despite differences in the degree of admixture in single populations, and overall more admixture between Troms and Lofoten (Fig. 2d). The main difference compared to K = 3 was that the isolated spring population from Lofoten (SL2) came out as a distinct group.

## Discussion

Overall, the genetic patterns among *Cochlearia* populations in Northern Norway support differentiation among ecotypes as previously suggested based on morphological and ecological investigations^16^. Preferences for different types of moist habitats are seen throughout the genus and local adaptation to divergent ecologies has probably been an important driver for speciation^12^.

The analyses suggest the beach ecotype as the ancestral *C*. *officinalis* ecotype in Northern Norway. This is supported by its intermediate position in the ordination plot, the relatively short branches in the network, and the high number of private markers. Also, the historical relationships, as displayed by the TREEMIX analysis, fit a scenario where the ancestral beach ecotype from the Lofoten area dispersed to Troms and in parallel locally adapted to the estuary and spring habitats. The Lofoten/Vesterålen area is one of the areas where the ice withdrew fairly early from Northern Scandinavia^29, 30^, supporting an early colonisation of *C*. *officinalis* from the south/southwest to this area.

Ecotypic differentiation in coastal versus inland habitats is found in many plant species^4, 5^. In the genus *Grindelia*, coastal, inland and intermediate ecotypes show similar levels of genetic differentiation to what we find in *C*. *officinalis* in Northern Norway^31^. Fragmented or patchy populations will potentially suffer from reduced gene flow between populations and increased genetic differentiation^32^. Whereas the exposed beach habitat of *C*. *officinalis* can be considered more or less continuous following the coastline, the estuary and spring habitats are typically more patchy and isolated. Spring vegetation types are thus described as “small islands in the landscape”^33^. The fragmentary nature of estuary and spring habitats, in combination with limited dispersal, can explain the patterns of strong population affiliation observed within each of the two sampling areas. Several species growing in coastal habitats have seeds that float well and are adapted to dispersal by sea currents over long distances^34^. In other coastal species, the seeds float less well and dispersal is dependent on the speed of the sea current^35, 36^. Dispersal of *Cochlearia* seeds has not been studied extensively, but they have no apparent adaptation for long distance dispersal and floating experiments indicate that dispersal with sea currents is limited to shorter distances^37, 38^. With putative limited ocean dispersal, it is not surprising that we find strong population affiliation of the *C*. *officinalis* populations and only limited gene exchange, primarily between geographically close populations.

The estuary habitat is connected to the sea, but still clearly separated from the more exposed habitat of the beach ecotype further out in the fjords. The streams might, however, lead to a more or less common seed pool. The *Cochlearia* plants in Northern Norway are obligate outcrossers^11^, and crossings between ecotypes resulted in seeds with high germination rate^16^. The distinctiveness of the beach and estuary ecotypes is probably related to selection to the rather special estuary habitat, which is characterised by brackish water conditions, regular inundation, and low levels of nutrient and organic material. Alternatively, differences in flowering time and temporal isolation between ecotypes could be a significant barrier to gene flow^5, 39^. In controlled experiments, plants of the estuary ecotype showed a tendency to delayed flowering compared to the beach ecotype^16^, but generally plants of both ecotypes have a prolonged flowering period throughout the whole summer and temporal isolation is less likely to explain the distinctiveness between plants in these two habitats.

Even though the estuary ecotype is morphologically the most distinct of the three ecotypes^16^, the spring ecotype turned out to be genetically the most distinct. Genetical distinctness is, however, related to degree of geographical isolation (Fig. S3), with the small isolated forest spring populations (Sørfjorddalen in Lofoten and Kvaløysletta in Troms) genetically most distinct, suggesting that drift may be an important force shaping the genetic patterns in this system. But also in this case, it is reasonable to assume that selection to the special spring habitat plays an important role for the distinct characteristics of the plants growing there. Compared to the exposed beaches and the often inundated estuary habitat, where an annual or biennial life history is most optimal, the spring habitat is much more sheltered and supportive of perenniality as usually found in the spring ecotype^16^.

Local adaptation occurs when a population evolves traits that support higher fitness in its native environment relative to populations from foreign environments^40^. Genetic differences between populations from contrasting environments can be indicative of selection for local adaptation, especially if these patterns are replicated. However, historic demographic events can generate similar patterns and ideally one should have fitness data from reciprocal transplant experiments to examine the genetics of local adaptation. In the absence of such data, combination of genetic differences and information about quantitative trait variation can be used as indirect evidence for the role of selection and may help to identify patterns of local adaptation^41^. Differentiation between forms can have occurred multiple times *in situ* (parallel evolution) or as a result of a single origin with subsequent dispersal to areas with suitable habitat^7^. In addition to the *Grindelia* example already mentioned^31^, other recent examples of parallel ecotypic differentiation have been shown in *Eucalyptus globules* ^39^ and *Senecio lautus* ^7^, with coastal ecotypes originating polytopically from more widespread, inland ecotypes. Our data, combined with previous morphological, ecological and eco-physiological studies of *C*. *officinalis*, build a good case of parallel ecotypic divergence as a result of repeated adaptation to the estuary and spring habitats. Analyses of both the SNP and the microsatellite data show differentiation among ecotypes, but also geographical separation within ecotypes, especially for the estuary and spring ecotypes which by the TREEMIX analysis are suggested to have originated polytopically from the ancestral beach ecotype. Cases of parallel evolution occurring within a species are important for understanding the interaction of natural selection, gene flow and geography on the origin of ecotypes. In our case, these factors or processes have most likely interplayed to produce the genetic patterns that we find among populations of the three ecotypes.

One of the intriguing challenges in ecological genomics is to identify the genes that underlie local adaptation. Cases of parallel ecotypic differentiation may provide particular good opportunities to search for candidate genes responding to natural selection, and allow for disentangling the effects of selection and drift. One common way to screen for adaptive loci is F_ST_-based outlier tests, which assume neutral genetic drift to affect the entire genome, whereas adaptive loci would be expected to show excess differentiation (outliers) among populations^42, 43^. A F_ST_-based outlier test of pairwise comparisons between *C*. *officinalis* ecotypes (beach vs. estuary and beach vs. spring) for each sampling area resulted in several candidate loci. Many of these are most likely the result of drift but for some outlier loci, we found a match between comparisons in Troms and Lofoten as would be expected if these are adaptive loci, or linked to adaptive loci that have evolved in parallel in the two areas. A couple of outlier loci found in both beach-estuary comparisons (Table 2), correspond indeed to genes (NHX1, GABA-T) that are known to be involved in salt tolerance in *Arabidopsis* and other plants, and could be important for adaptation to brackish conditions^44–47^. In most cases, the traits that confer local adaptations are polygenic quantitative traits^41^, and identification of loci that govern variation in such traits is a challenging task and will require a genomic region-based approach that can detect genetic hitchhiking regions^48^. Further, the common isolating traits acquired in different populations as a result of parallel ecotypic differentiation may not necessarily be governed by the same mutation, gene or even pathway in different replicates^49^. In any case, identification of genes that play a role in adaptation will require selection experiments in controlled and field environments to directly measure their effects on fitness, and in addition functional gene analyses to detect loci that actually alter fitness^7, 48^.

As RADseq is only a representation of the genome, important regions of the genome, and thus also several loci potentially involved in adaptive divergence, are most likely overlooked^50, 51^. Lowry *et al*.^27^ estimated the median density of markers from recent studies performing genome scans with RADseq to be 4.08 RADtag per megabase. With haplotypes being one to three orders of magnitude shorter for many species, they concluded that RADseq will miss many loci under selection. With a density of marker estimation of 7.26 RAD loci/Mbp for the current study, we have probably only been able to identify a minor portion of the actual number of markers that could be under selection as part of the diversification between the ecotypes of *C*. *officinalis*. However even with the limitations of the current approach (RADseq), we were able to identify ecologically-relevant loci that could be involved in divergent adaptation.

Determining the genes and the genetic architecture of traits involved in adaptive divergence between ecotypes is crucial to understand the process of speciation. A common intermediate stage in the process towards reproductive isolation is the evolution of partially reproductively isolated ecotypes, resulting from adaptation to different habitats. Despite being isolated ecologically, or partly so, the *Cochlearia* ecotypes still represent a quite early stage in this process where reproductive isolation has not yet evolved. Whether or not the ecotypes may eventually become fully distinct species, studying them may give us the opportunity to observe the processes leading to diversification. In already diverged and well diagnosed species, these processes are usually even more obscured. Young incipient species are more likely to display signatures of selective sweeps that can point to asymmetry in selection between habitats^52^. Sequenced annotated genomes would open up for this and also for detailed investigations regarding the possible link between autopolyploidy and rapid phenotypic diversification. In this light, the autotetraploid *Cochlearia* in Northern Norway represents an interesting example of parallel ecotypic divergence, illustrated by non-random organisation of genetic variation across the landscape that may, or may not, in time become reproductively isolated species.

## Materials and Methods

### Plant Material and DNA extraction

Two populations of each of the three *Cochlearia* ecotypes were collected from each of the two areas in Northern Norway: Troms and Lofoten (Table 1, Fig. 2). From each population, leaf tissue of 10 individuals was dried and stored in silica gel. When available, mature seeds were also sampled, preferably from the same individuals. Five representative individuals from each population were collected as herbarium vouchers and deposited at the herbarium of the Natural History Museum, University of Oslo (O). To obtain fresh leaf tissue for flow cytometry, seeds were germinated and plants grown in controlled growth chambers at the University of Oslo (18 h light at 18 °C; 6 h dark at 10 °C). To confirm that the sampled plants were tetraploids, representative plants from all populations (altogether 54 individuals, Table 1) were analysed by flow cytometry to obtain relative nuclear DNA amounts (see Supporting Information Methods S1 for further details on how the flow cytometry analyses were performed).

DNA was extracted from c. 30 mg silica-dried leaf tissue from each individual with the E.Z.N.A. SP Plant DNA Kit (Omega bio-tek), following the protocol for dry samples with minor modifications. Before extraction, the samples were crushed for 1–2 min at 20 Hz with two 3 mm tungsten carbide beads in a tissuelyser Retsch MM301 (Qiagen). In most cases, elution with 50 µl (run through once or twice) was used. Before RADseq, the DNA samples were cleaned with NucleoSpin gDNA Clean-up (Macherey-Nagel).

### RADseq analyses

RADseq libraries were prepared by single digest reactions using *Pst*I, combinatorial inline barcoding, and size selection with magnetic beads. The protocol was adapted from previous studies^53, 54^, with modifications as indicated below. Altogether 120 *Cochlearia* samples were included in two libraries (60 samples in each); of these 91 (89 individuals and two library replicates), representing the 12 populations from Northern Norway, were included in the data analyses for this study. For each individual, 125 ng DNA was digested at 37 °C for 2 h with 15 U *Pst*I-HF (NEB). To remove *Pst*I-HF (which cannot be heat inactivated), all samples were cleaned using SPRIselect Reagent Kit (Beckman Coulter) with no size selection. After ligation of P5 adapters, samples with different P5 barcodes were pooled together in five sublibraries and sheared by sonication using a Bioruptor Pico (Diagenode) with three cycles of 45 s “on” and 60 s “off” at 4 °C to achieve an average size of c. 400 bp. Samples were purified with MinElute Reaction Cleanup Kit (Qiagen) followed by left (0.7x) and right (0.55x) side size selection with SPRIselect Reagent Kit. After ligation of P7 adapters, similar cleaning and size selection, this time only on the left side (0.65x), were performed both before and after PCR amplification with Phusion Master Mix (NEB). The libraries were sent to paired-end sequencing, each in one Illumina HiSeq2000/HiSeq2500 lane (100 bp/125 bp) at the Norwegian Sequencing Centre, Oslo, Norway (http://www.sequencing.uio.no/).

Raw Illumina reads were processed with STACKS v. 1.23^55, 56^. To demultiplex the individuals and remove low quality data, the program *process_radtags* was run with the following settings: *Pst*I as restriction enzyme, removal of any read with an uncalled base, discarding reads with low quality scores, and rescuing barcodes and RADtags. After reads from the two libraries were cut to the same length (94 bp), *ustacks*, *cstacks* and *sstacks* were run with only forward reads. Different values for *m* (minimum number of identical raw reads required to create a stack), *M* (number of mismatches allowed between loci when processing a single individual) and *n* (number of mismatches allowed between loci when building the catalog) were tested to find the settings that maximised the number of reliable loci identified from the reads (see Supporting Information Methods S2 for further details). The settings used in the end were *m* = 3, *M* = 4 and *n* = 1. To further optimise the pipeline for tetraploids, each individual was allowed to have four alleles (plus one extra to account for potential sequencing errors) by setting the–max_locus_stacks to 5 (default is 3 when expecting diploids). The *export_sql*.*pl* script was used to create a whitelist of loci that contained 1–10 SNPs (snps_l = 1 and snps_u = 10). The program *populations* was used to link the individuals to their respective population and to produce structure-, vcf-, phylip- and haplotype files, each optimised for a specific purpose. Except for the haplotype file, only one SNP per locus was retained (i.e. the first SNP on each locus) to minimise as much as possible linkage of markers. STACKS is, at least at this point, unable to write out full polyploid genotypes, hence our final filtered datasets were diploid-like. A great majority of SNPs are, however, expected to be bi-allelic at the population level, meaning that it is only information about partial heterozygotes which is lost. Information about the filters used (percentage of individuals and populations required for a locus to be processed) and the number of SNPs obtained in each case can be found in Supporting Information Table S1. As replicated samples clustered together in the initial data analyses, only one per accession was included in the final analyses. The vcf file was converted to the appropriate format with PGDSpider v. 2.0.8.2^57^ for analyses done in GENEPOP v. 4.2^58, 59^ and ARLEQUIN v. 3.5.2.2^60^.

The number of private alleles and the inbreeding coefficient (F_IS_) for ecotypes and single populations were obtained from running the program *populations* in STACKS. GENEPOP was used to calculate the number of migrants based on private alleles (Nm) for pairwise comparisons of single populations (corrected for size). Violin plots summarising Nm values were constructed using the library vioplot (available from https://CRAN.R-project.org/package=vioplot) in R v. 3.1.2. AMOVAs were run in ARLEQUIN to estimate genetic differentiation among populations and among higher level groups (ecotypes and geographical areas). Analyses using ecotypes were run separately for the two geographical areas. The analysis for Lofoten was done both with and without the spring population SL1 (Himmeltind), as this population turned out to be genetically more similar to the beach populations than to the other spring populations.

The structure file was used to perform a PCA with the R package *adegenet* v. 1.4–1^61, 62^ in R v. 3.2.5^63^ using allele frequencies centred to mean zero and scaled, missing values treated as zero, and Euclidean as distance measure. One individual (MKB12–4–17) had a slight outlier position and was removed in the final PCA to allow for better resolution of the remaining individuals. Population structure was further investigated with STRUCTURE v. 2.3.3^64^ using the admixture model and correlated frequencies. A tetraploid input file was constructed by using the recessive allele option^65^ and ploidy set to four to allow for ambiguity in partial polyploid heterozygotes^66^. The analysis was run with K = 1–13, 10 runs for each K, 1 million iterations and burn-in of 100,000 using the Lifeportal at the University of Oslo (https://lifeportal.uio.no/). Results were summarised in STRUCTURE HARVESTER web v. 0.9.94^67^ and CLUMPAK beta v.^68^, producing likelihood and deltaK graphs^69^. The optimal number of groups converged to the same solution for all replicate runs (confirmed by inspecting the plots) and was visualised using DISTRUCT v. 1.1^70^ and as pie charts on a map of Northern Norway using QGIS v. 2.4.0^71^. The map layer was extracted from GADM version 1.0^72^.

The phylip file was used to produce a phylogenetic network in SPLITSTREE4^73^. Splits were created from Jukes Cantor distances and visualised as a neighbour net with each end node representing an individual. TREEMIX v. 1.12^74^ was used to address historical relationships between populations. Using VCF-tools v. 0.1.12^75^ and PLINK v. 1.90^76^, the vcf file was converted to a frequency file that could be transformed to a treemix file using the *plink2treemix* script available for TREEMIX (https://bitbucket.org/nygcresearch/treemix/downloads). TREEMIX was run with a Scottish *C*. *officinalis* population from Aberdeenshire (included in the RADseq libraries) as outgroup, visualised in R and illustrated in combination with the STRUCTURE pie charts. The number of migration events was tested by starting at zero and adding one by one until the residual plot stopped improving.

To detect possible RADseq loci under selection, BAYESCAN v. 2.1^42, 43, 77^ was used with default settings. The haplotype file produced from *populations* was used together with a python script to create the input file (containing haplotype information) for BAYESCAN^78^. Ecotypes were tested in pairwise comparisons between the likely ancestral ecotype (beach) and the estuary and spring ecotypes, respectively. Tests were performed for each geographical area (Troms and Lofoten) separately and then compared to look for possible common outlier loci. The program *sort_read_pairs*.*pl* in STACKS was used to collect the reverse reads (the read pairs) of the outlier loci, and of 1,000 random loci of the catalog in order to construct a reference set for further enrichment analyses. The program *exec_velvet*.*pl* in STACKS was used to extend the contigs of the outlier and reference loci. The outlier loci for each comparison and the reference set were annotated and used for further GO enrichment analyses in BLAST2GO v.3.2.7^79^. Fisher’s exact tests were implemented at a threshold p-value of 0.05. The enriched GO terms from each comparison were summarised, applying thinning based on semantic similarity, and visualised with REViGO^80^.

The STACKS-pipeline as well as TREEMIX and BAYESCAN analyses were run on the Abel cluster, owned by the University of Oslo and the Norwegian metacentre for High Performance Computing (NOTUR).

### Microsatellite analysis

The M13-tailing approach from Schuelke^81^ was used to test twenty primers developed for other Brassicaceae taxa (*Arabidopsis*, *Brassica* and *Draba*; Supporting Information Table S2) for co-amplification in 15 *Cochlearia* individuals. Six microsatellites successfully amplified and were used to analyse 10 individuals from each of the 12 *Cochlearia* populations from Northern Norway, following the protocol by Vik *et al*.^82^ except that 10 μl PCR reaction volumes were used. The annealing temperature used for each microsatellite after optimisation is given in Supporting Information Table S6. At least five replicates and one negative control were included per 96-well plate.

Microsatellite genotypes (based on allele sizes) were assessed in GENEMAPPER v. 3.7 (Life Technologies/Applied Biosystems). The automated scoring was manually edited to make sure that the scoring was plausible, i.e. tetraploids had not more than four alleles, and replicates had identically scored profiles. The R package POLYSAT v. 1.3^83^ was used to construct a tetraploid input file, allowing ambiguity in partial heterozygotes, which was analysed in STRUCTURE with the same settings as for the RADseq data. F_IS_ was calculated with SPAGeDI (Spatial Pattern Analysis of Genetic Diversity) that offers a way to estimate the allele frequencies in polyploids by assuming that each of the alleles in a partial heterozygote has an equal likelihood of being present more than once^66^.

## Electronic supplementary material

Supporting Information
