# Supplementary material for: RADseq provides evidence for parallel ecotypic divergence in the autotetraploid Cochlearia officinalis in Northern Norway
Source: Sci Rep. 2017 Jul 17;7:5573. doi: 10.1038/s41598-017-05794-z (PMC5514025; doi:10.1038/s41598-017-05794-z)
Supplement: Supplementary file 1 — Supporting Information [file 41598_2017_5794_MOESM1_ESM.pdf]

Supporting Information

RADseq provides evidence for parallel ecotypic divergence in the autotetraploid *Cochlearia officinalis* in Northern Norway

Marie K. Brandrud[1,2,*], Ovidiu Paun[2], Maria T. Lorenzo[2], Inger Nordal[3] and Anne K. Brysting[1]

[1]Centre for Ecological and Evolutionary Synthesis, Department of Biosciences, University of Oslo, 0316 Oslo, Norway

[2]Department of Botany and Biodiversity Research, University of Vienna, 1030 Vienna, Austria

[3]Department of Biosciences, University of Oslo, 0316 Oslo, Norway

[*]*marie.kristine.brandrud@univie.ac.at*

Figure S1: Habit and habitat of the three ecotypes of autotetraploid *Cochlearia officinalis.*

Figure S2: Violin plots of the distribution of number of migrants (Nm) based on RADseq data.

Figure S3: DeltaK and likelihood of K for STRUCTURE analysis of RADseq data (K = 2 and K = 9 are visualized).

Figure S4: BAYESCAN and BLAST2GO results from RADseq data, testing the beach and estuary ecotypes within Troms and Lofoten.

Figure S5: BAYESCAN and BLAST2GO results from RADseq data, testing the beach and spring ecotypes within Troms and Lofoten.

Figure S6: DeltaK and likelihood of K for STRUCTURE analysis of microsatellite allele sizes (K = 3 and K = 7 are visualized).

Table S1: Information about input files used in downstream analyses of RADseq data.

Table S2: Private alleles and inbreeding coefficient (F_{IS}) based on RADseq data.

Table S3: Number of migrants (Nm) based on RADseq data.

Table S4 Analysis of molecular variance (AMOVA) based on RADseq data.

Table S5: BAYESCAN outlier loci from RADseq data.

Table S6: Information about microsatellite primers.

Methods S1: Flow cytometry methodology.

Methods S2: Selecting settings for maximizing number of reliable RADseq loci.

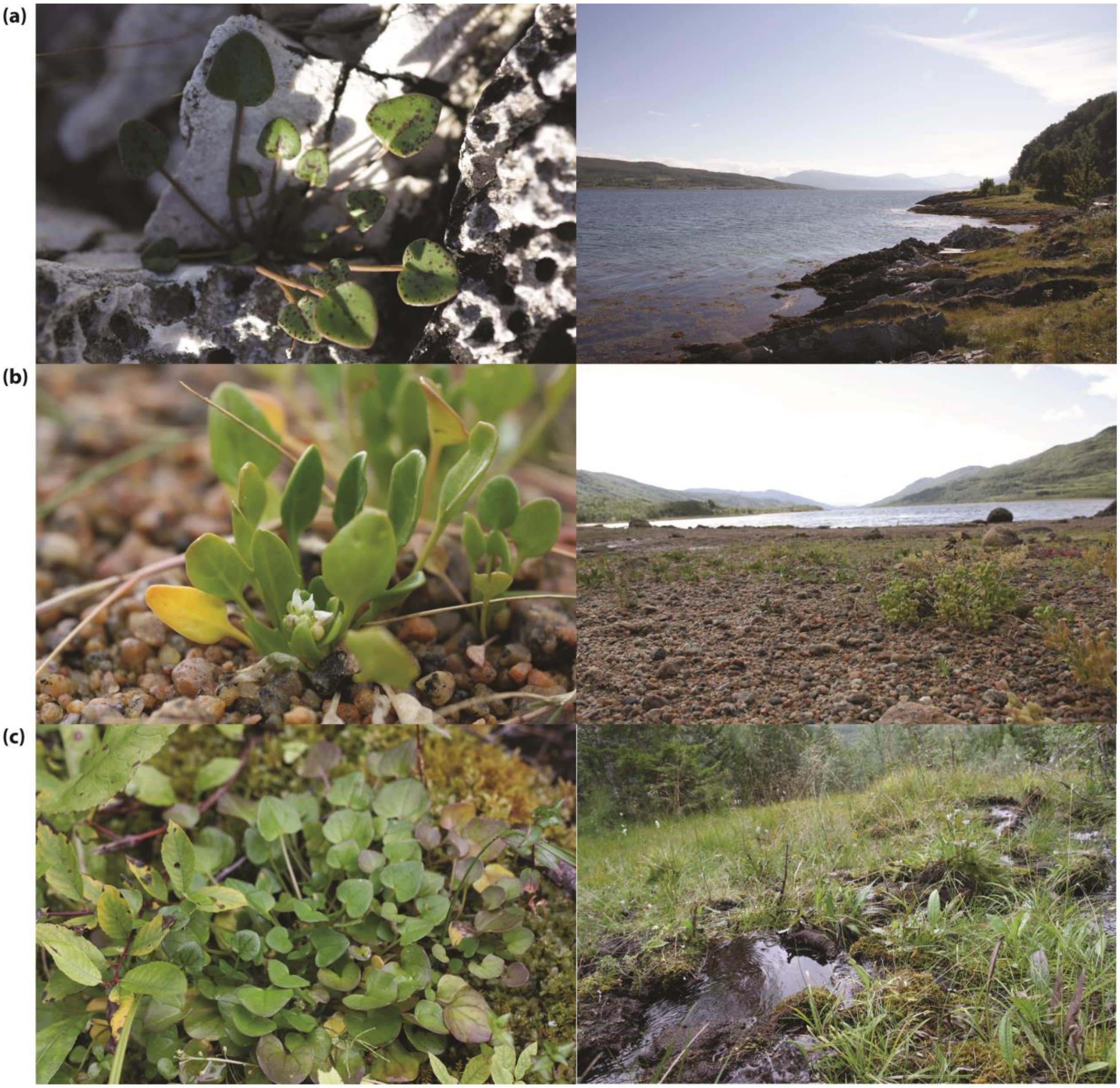

Figure S1. Habit and habitat of the three ecotypes of *Cochlearia officinalis* (2*n* = 24) in Lofoten, Norway. (**a**) the beach ecotype (ssp. *officinalis*) at locality Tjeldsundet, (**b**) the estuary ecotype (ssp. *norvegica*) at locality Kanstadbotnen, and (**c**) the spring ecotype (ssp. *integrifolia*) at locality Sørfjorddalen. (Photo: M.K. Brandrud).

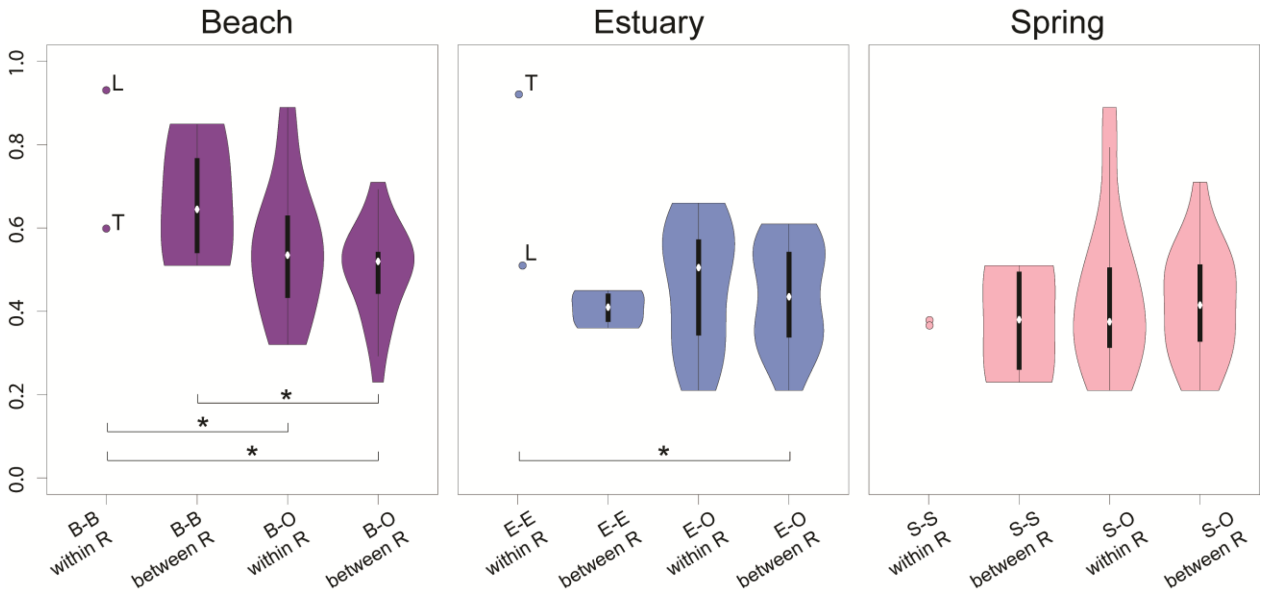

Figure S2. Violin plots of the distribution of number of migrants (Nm) per population pair. The calculations are based on private alleles, from 5,329 SNPs from RADseq data. For each of three ecotypes analysed, the values are categorized as within ecotype within a region, within an ecotype between regions, from one ecotype to the others within regions and between regions. For the within ecotype within a region comparison, only two values are available for each ecotype, which are shown as single points instead of violin plots. The level of significance of the difference in distribution was tested with unpaired t-tests in R, and significantly different pairs (i.e., $p < 0.05$) are represented by *. Populations are named according to ecotype (B - beach ecotype, E - estuary, S - spring) and geography (T - Troms, L - Lofoten), see Table 1. O - others, R - region.

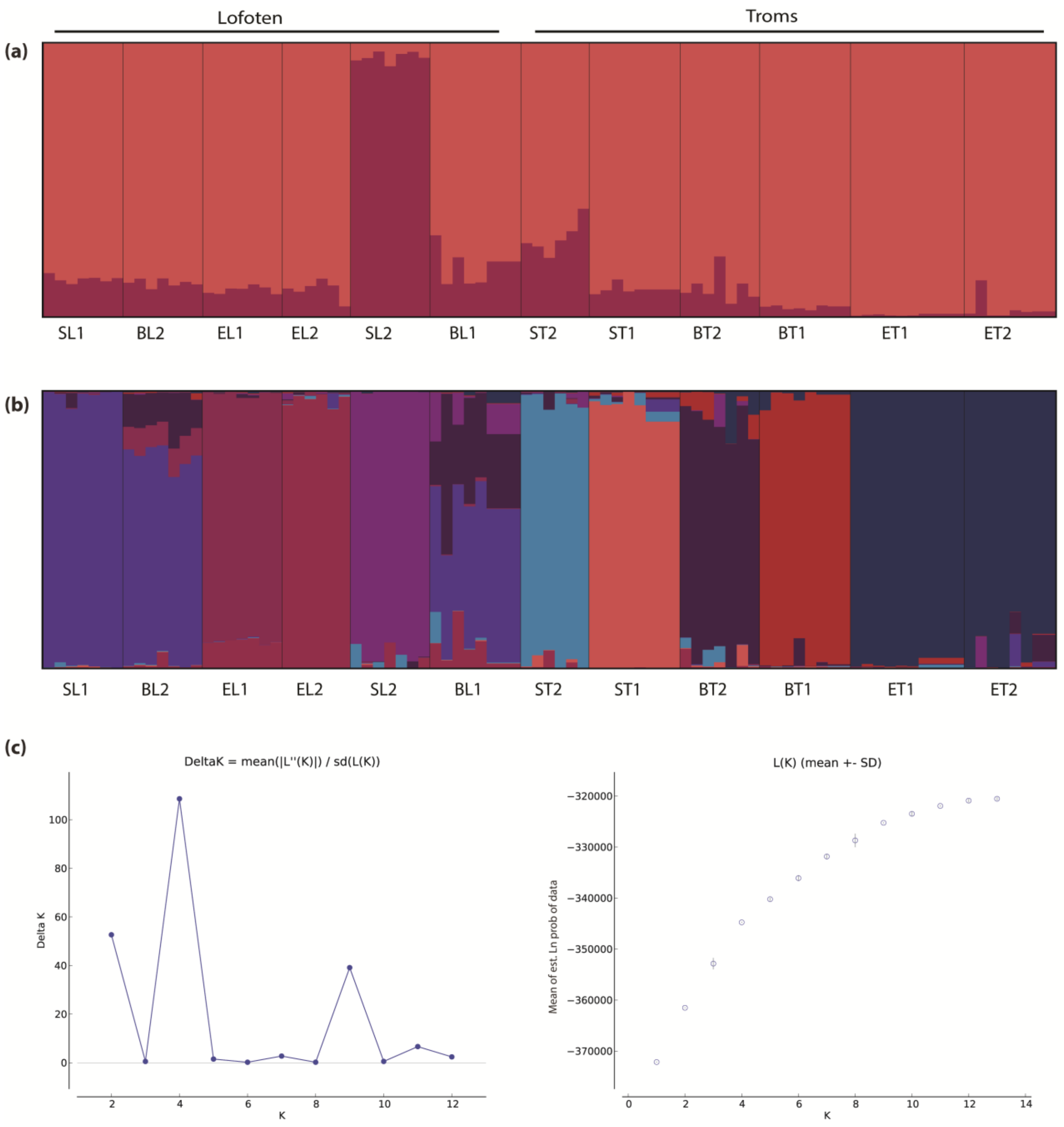

Figure S3. Results from STRUCTURE analysis. K = 2 (**a**) and K = 9 (**b**) of 89 individuals and 12 populations of *Cochlearia officinalis*, based on 4,296 SNPs from RADseq data. Each individual is represented by a bar and colours represent the proportional assignment to the STRUCTURE groups. Populations are separated by a black line and named according to ecotype (B = beach, E = estuary, S = spring) and geography (T = Troms, L = Lofoten), see Table 1. (c) DeltaK (left) and mean likelihood of K (right).

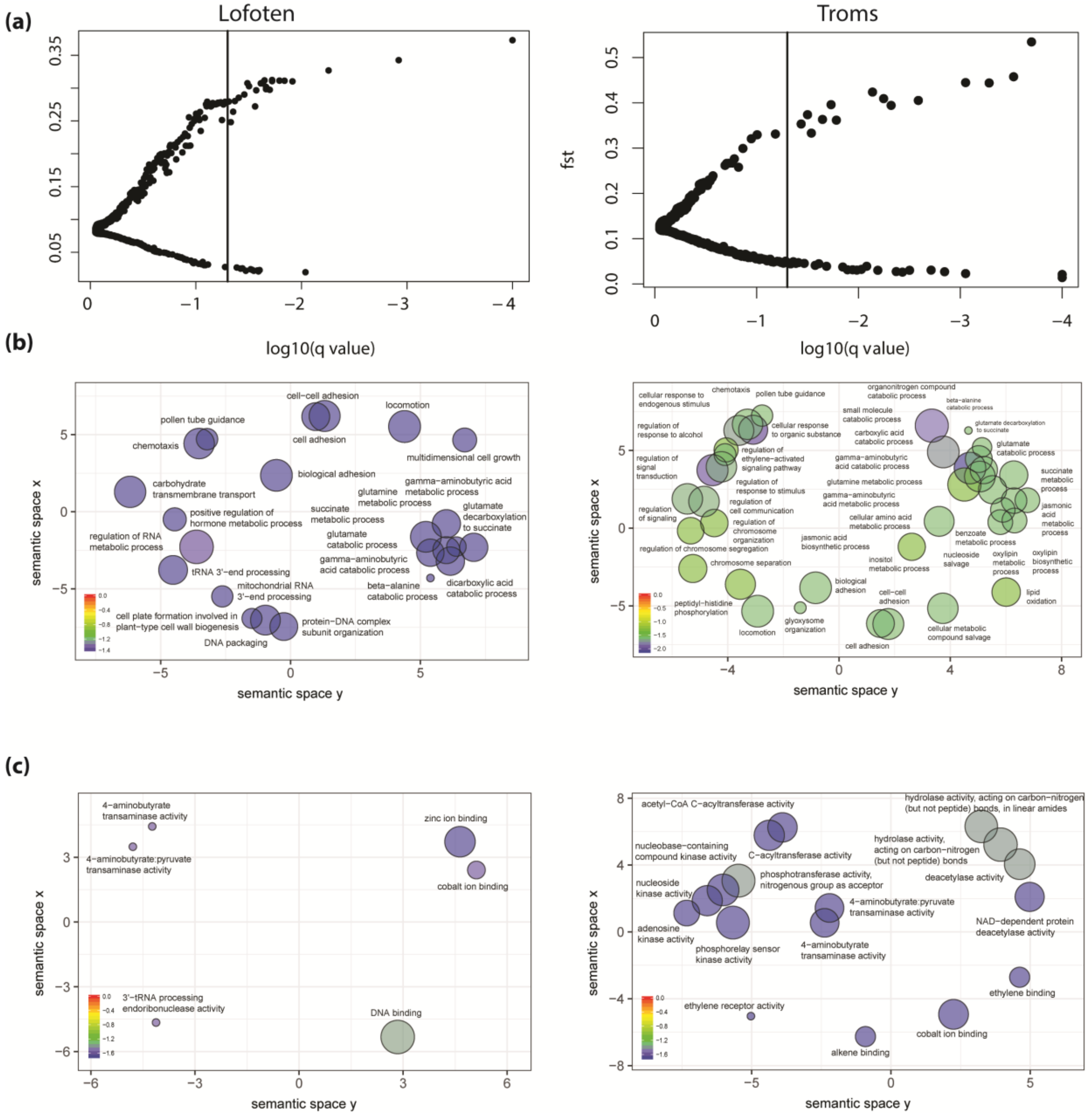

Figure S4. BAYESCAN and BLAST2GO results from RADseq data. (**a**) Results from BAYESCAN analysis testing for outliers between the beach and estuary ecotype. Annotation information about the three outliers that were found in both comparisons (Lofoten and Troms) is given in Table 2. (**b**) Enriched biological processes ($p < 0.05$) for the outliers between the beach and estuary ecotype, visualized as revigo plots from the BLAST2GO results. (**c**) Enriched molecular functions ($p < 0.05$) for the outliers between the beach and estuary ecotype, visualized as revigo plots from the BLAST2GO results. Bubble size is proportional to the frequency of the respective term in the public GO database. The colour represents the log10 value of the significance of the Fisher's tests for enrichment, corresponding to the indicated scale.

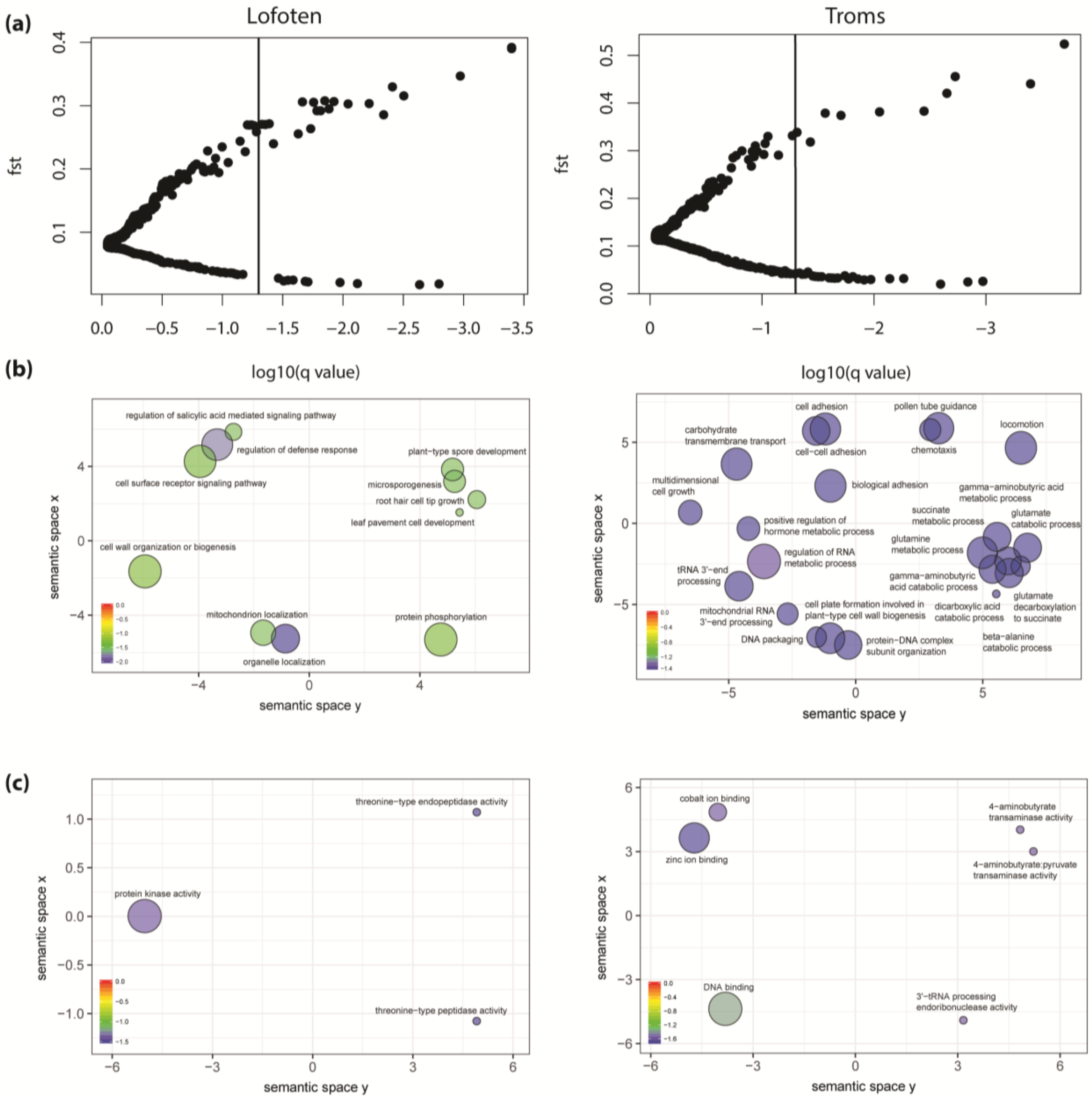

Figure S5. BAYESCAN and BLAST2GO results from RADseq data. (**a**) Results from BAYESCAN analysis testing for outliers between the beach and spring ecotype. Annotation information about the four outliers that were found in both comparisons (Lofoten and Troms) is given in Table 2. (**b**) Enriched biological processes ($p < 0.05$) for the outliers between the beach and spring ecotype, visualized as revigo plots from the BLAST2GO results. (**c**) Enriched molecular functions ($p < 0.05$) for the outliers between the beach and spring ecotype, visualized as revigo plots from the BLAST2GO results. Bubble size is proportional to the frequency of the respective term in the public GO database. The colour represents the log10 value of the significance of the Fisher's tests for enrichment, corresponding to the indicated scale.

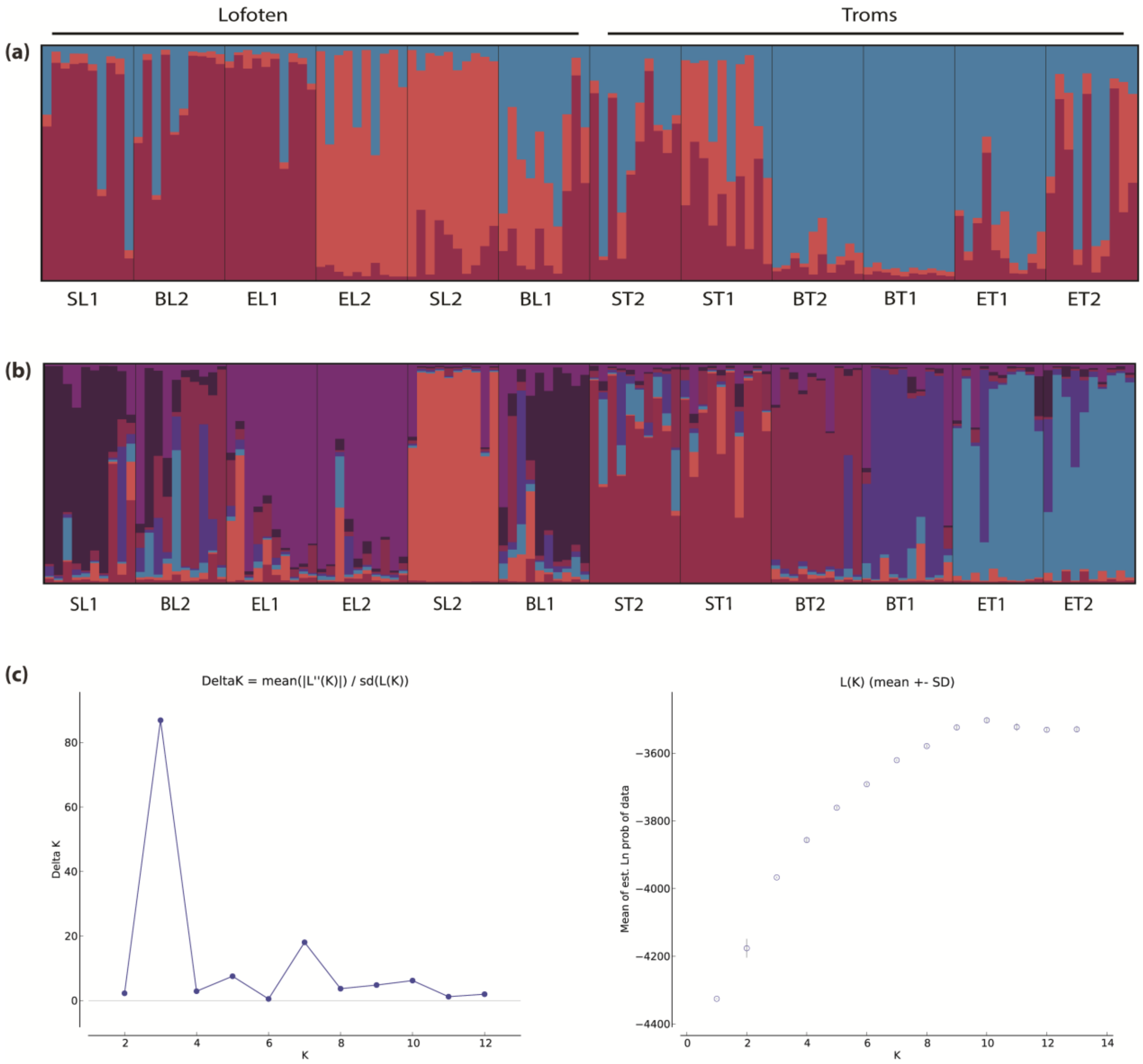

Figure S6. Results from STRUCTURE analysis. K=3 (**a**) and K=7 (**b**) of 120 individuals and 12 populations of *Cochlearia officinalis*, using microsatellite allele sizes. Each individual is represented by a bar and colours represent the proportional assignment to the STRUCTURE groups. Populations are separated by a black line and named according to ecotype (B = beach, E = estuary, S = spring) and geography (T = Troms, L = Lofoten), see Table 1. (**c**) DeltaK (left) and mean likelihood of K (right).

Input file	-r	-p	Populations/individuals included	Number of SNPs (loci)	Program
phylip	100%	-	89 individuals	4311	SPLITSTREE4
structure	100%	100%	12 populations (89 individuals)	4296	ADEGENET<br>STRUCTURE
vcf	100%	90%	12 populations<br>+ root (95 individuals)	5982	TREEMIX
vcf	100%	90%	12 populations (89 individuals)	5329	ARLEQUIN<br>GENEPOP
haplotype	80%	75%	4 populations (31 individuals)<br>beach + estuary from Lofoten	27459<br>(16151)	BAYESCAN
haplotype	80%	75%	4 populations (30 individuals)<br>beach + spring from Lofoten	26408<br>(15172)	BAYESCAN
haplotype	80%	75%	4 populations (29 individuals)<br>beach + estuary from Troms	24782<br>(15775)	BAYESCAN
haplotype	80%	75%	4 populations (28 individuals)<br>beach + spring from Troms	24377<br>(15472	BAYESCAN

Table S1. Information about input files used in downstream analyses of RADseq data. The filters used in the STACKS program *population*: -r: minimum individuals in a population required to process a locus for that population (given as percentage). -p: minimum number of populations in which a locus must be present to process a locus (given as percentage). In all cases, only loci that contained 1-10 SNPs were included (-W: giving a whitelist of which loci to allow), and for the four first files only one SNP per locus was selected (--write_single_snp). For each file, information about the number of populations/individuals included and amount of SNPs obtained in each case, as well as the programs for which the files were used as input files, are given.

Ecotype	Population	Private alleles	Inbreeding coefficient (F_{IS}) RADseq	Inbreeding coefficient (F_{IS}) microsatellite
Beach		661		
Estuary		603		
Spring		494		
Beach	BL1	122	-0.0840	0.031
	BL2	257	-0.1261	0.116
	BT1	73	-0.1182	0.052
	BT2	148	-0.1146	-0.051
Estuary	EL1	135	-0.1185	-0.119
	EL2	130	-0.1098	-0.252
	ET1	75	-0.1076	0.138
	ET2	201	-0.0997	-0.184
Spring	SL1	208	-0.1092	-0.066
	SL2	49	-0.0705	0.005
	ST1	117	-0.1093	-0.214
	ST2	79	-0.0927	0.071

Table S2. Number of private alleles and and inbreeding coefficient (F_{IS}) for *Cochlearia officinalis* ecotypes and single populations. The calculations for private alleles are based on SNPs from RADseq data. The inbreeding coefficients are calculated on SNPs from RADseq and on microsatellites alleles independently. Populations are named according to ecotype (B = beach, E = estuary, S = spring) and geography (T = Troms, L = Lofoten), see Table 1.

Within Troms

POP ID	BT1	BT2	ET1	ET2	ST1	ST2
**BT1**	0.00	0.59	0.41	0.51	0.33	0.32
**BT2**		0.00	0.55	0.66	0.50	0.52
**ET1**			0.00	0.93	0.28	0.29
**ET2**				0.00	0.36	0.37
**ST1**					0.00	0.37
**ST2**						0.00

Within Lofoten

POP ID	BL1	BL2	EL1	EL2	SL1	SL2
**BL1**	0.00	0.94	0.58	0.57	0.81	0.44
**BL2**		0.00	0.66	0.62	0.89	0.38
**EL1**			0.00	0.51	0.57	0.27
**EL2**				0.00	0.50	0.21
**SL1**					0.00	0.38
**SL2**						0.00

Between Troms and Lofoten

POP ID	BL1	BL2	EL1	EL2	SL1	SL2
**BT1**	0.51	0.55	0.39	0.36	0.49	0.23
**BT2**	0.85	0.74	0.54	0.55	0.71	0.36
**ET1**	0.54	0.52	0.38	0.36	0.48	0.21
**ET2**	0.61	0.60	0.45	0.44	0.56	0.29
**ST1**	0.46	0.53	0.33	0.32	0.49	0.23
**ST2**	0.51	0.52	0.34	0.37	0.51	0.27

Table S3. Number of migrants (Nm) based on private alleles for *Cochlearia officinalis*. The calculations are based on 5,329 SNPs from RADseq data for 89 individuals and 12 populations. Populations are named according to ecotype (B = beach, E = estuary, S = spring) and geography (T = Troms, L = Lofoten), see Table 1.

Source of variation	d.f.	Sum of squares	Variance components	Percentage variance
**Geography**				
Among groups	1	973.455	4.12002	1.9
Among populations within groups	10	5924.819	26.88982	12.4
Within populations	170	31590.453	185.82619	85.7
**Ecotypes within Troms**				
Among groups	2	1992.155	13.90747	5.23
Among populations within groups	3	1759.061	24.53048	9.22
Within populations	82	18668.42	227.1016	85.56
**Ecotypes within Lofoten (without SL1)**				
Among groups	2	1266.448	10.15599	5.75
Among populations within groups	2	737.554	14.34924	8.12
Within populations	73	11111.485	152.21212	86.13
**Ecotypes within Lofoten (with SL1)**				
Among groups	2	1505.851	2.86409	1.15
Among populations within groups	3	1948.999	28.09209	11.32
Within populations	88	19112.682	217.18957	87.53

Table S4. Analysis of molecular variance (AMOVA) of *Cochlearia officinalis* populations. The calculations are based on SNPs from RADseq data. Geography as higher level groups: the two sampling areas, Troms and Lofoten. Ecotype as higher level groups: beach, estuary, and spring. The analysis for Lofoten was done both with and without the spring population SL1 (Himmeltind).

**Beach-Estuary**		**Beach-Spring**	
**Lofoten**	**Troms**	**Lofoten**	**Troms**
47	729	1279	765
1818	1533	2010	2457
3105	1893	**2851***	**2851***
3620	2457	**2873***	**2873***
5589	2967	4551	3196
7397	3196	4872	5291
11000	3303	5012	7341
11412	3968	**8172***	**8172***
**11990***	4386	11178	10288
**13110***	4727	12996	10502
16709	5902	13871	10812
**16828**	5903	14904	**13110**
17740	7205	15873	13358
**18967***	7366	16082	13564
**19348**	10288	**16828**	18424
20586	10877	17539	**18967**
21394	11903	**19348***	19028
22340	**11990***	19378	**19348***
22678	**13110***	22092	19436
23117	13260	22340	**20686**
23202	13502	23580	21584
24100	14095	24445	31569
27328	18120	24703	32621
29586	18377	25593	33460
31659	**18967***	26256	34481
34315	**20686**	26791	35513
35900	20911	28675	36332
37929	22579	28744	45973
41970	22979	30356	47796
42163	23575	32058	48206
42827	28080	37554	51616
45845	31740		57091
46632	36103		
47603	39313		
51752	44160		
56426	45912		
	49767		
	59780		

Table S5: Outlier loci from BAYESCAN analysis of *Cochlearia officinalis* populations. The analyses are based on SNPs from RADseq data, and are done for beach-estuary and beach-spring, comparisons, respectively. Loci occurring in more than one comparison are higlighted by bold. For loci labelled with an asterisk, annotation information is given in Table 3.

Name	T_a	Forward primer	Reverse primer	Fragment length
*DnA222[1]	48	GTGGCAATTTGCTTCCAACC	GCGCAGTGAGATGGATTTCTGG	142-144
*DnB101[1]	48	TGGCTTACCATTGCTGTCC	CCGCATTGTGTTGTTCTTG	123-288
DnB207[1]	-	GGACGGCTGCATTTTCAC	TCAGCTTCACACCAAACAATTC	
DnB220[1]	-	GCAAAGCAGAGCGTAGAATGG	ACTCGGACGTCTCAATCAGC	
*AthCTRI[2]	51	TATCAACAGAAACGCACCGAG	CCACTTGTTTCTCTCTCTAG	135-143
*AthSO392[3]	51	GTTGATCGCAGCTTGATAAGC	TTGGAGTTAGACACGGATCTG	148-203
BRMS008[4]	-	AGGACACCAGGCACCATATA	CATTGTTGTCTTGGGAGAGC	
AthGAPAb[5]	51	CACCATGGCTTCGGTTACTT	TCCTGAGAATTCAGTGAAACCC	
BRMS033[4]	51	GCGGAAACGAACACTCCTCCCATGT	CCTCCTTGTGCTTTCCCTGGAGACG	
BRMS037[4]	-	CTGCTCGCATTTTTTATCATAC	TACGCTTGGGAGAGAAAACTAT	
*MR187[6]	51	GAGTTTTGGTTCCACCATTA	CCCTTCAGCCTTTGATAAAT	143-243
SSL2[5]	51	CATGTACTGGGATTCAGTGTCC	CGTCCTTTGTGTGGTTACACG	
nga129[3]	-	TCAGGAGGAACTAAAGTGAGGG	CACACTGAAGATGGTCTTGAGG	
AthSO191[3]	-	TGATGTTGATGGAGATGGTCA	CTCCACCAATCATGCAAATG	
DnB123[1]	-	CAGTGCAAAATGCGTGAAT	GCGTGGAGATAGAGAAAGAGC	
DnB106[1]	-	TGCGCGCAGAGACAAAGGAG	GAATCCGCCATAGCCGAGGTTG	
DnA8[1]	-	CTTTGGTGGTCTTCCTTG	ATACGATTCCGAGTATTACCTC	
DnB3[1]	-	GCCGTTGTATTGTAGAGTGAG	ACTGGGTCCTCGCTAAAC	
*DnA117[1]	48	TTGTATTCATCGGTTGTGTATC	ACCTGGAAGCACTGGTTC	232-242
DnA138[1]	-	CTTCCTGCGACATCACTCAAAC	TACGGATTGGAGAGAATTCTGAGC	

Table S6. Microsatellite primers developed for other Brassicaceae species and tested for cross-amplification in *Cochlearia*. Primers were selected based on Skrede *et al.*[1], but reference to the original publications are given for all tested primers. Primers marked with an asterisk were used in the final microsatellite analysis of 120 tetraploid *C. officinalis* individuals from Northern Norway. Annealing temperature (Ta) is given for primers resulting in successful amplification. Fragment length is given for the microsatellites that were scorable in GENEMAPPER.

Methods S1

Flow cytometry

Relative fluorescence intensities were estimated by flow cytometry. For 42 individuals representing six populations from Troms, fresh leaves were analysed by Plant Cytometry Services (The Netherlands). For each individual 1-2 cm^2/50-100 mg leaf tissue was cut in ice-cold buffer to isolate and dye the nuclei from the plant cells. The buffer, slightly modified from Arumuganathan & Earle[7], contained 5.0 mM Hepes, 10.0 mM $MgSO_4 \cdot 7\ H_2O$, 50.0 mM KCl, 0.2% Triton X-100, 0.1% DTT (dithiothreitol), 1.0% PVP-40, and 2 mg/l DAPI (4',6-diamino-2-phenylindol), at pH 7.5. The suspension was sent through a nylon filter with mesh size 50 µm and then passed through a CyFlow Space flow cytometer (Partec GmbH, Münster, Germany) with a UV high power led (365 nm) lamp. No internal standard was used, but presumed diploid (*C. aestuaria* Lloyd Heywood) from northern Spain and octoploid (*C. anglica* L.) individuals from southern Sweden were also analysed to be able to compare relative DNA amounts.

For 12 individuals representing six populations from Lofoten, silica-dried leaves were analysed by Pavel Trávníček (Academy of Sciences of the Czech Republic), using the two-step methodology according to Doležel *et al.*[8]. *Solanum pseudocapsicum* L. (2C = 2.58 pg) was used as internal standard[9]. Usually 0.5 cm^2 leaf tissue of a *Cochlearia* sample and an appropriate amount of internal reference standard were chopped with razor blade in 0.5 ml of ice-cold Otto I buffer (0.1 M citric acid, 0.5% Tween 20). A crude suspension of nuclei was filtered through a nylon mesh (loop size 0.42 µm), incubated in room temperature for at least 5 min and stained with 1 ml of Otto II buffer (0.4 M $Na_2HPO_4 \cdot 12\ H_2O$) supplemented by DAPI and 2-mercaptoethanol in final concentrations of 4 µg/ml and 2µl/ml, respectively. After short incubation (5 min) in room temperature, relative fluorescence intensity of at least 3000 nuclei was recorded using a Partec CyFlow Space flow cytometer (Partec) with a UV high power led (365 nm) lamp. Presumed diploid (*C. aestuaria*) individuals from northern Spain were also analysed for comparison.

Methods S2

Processing of RADseq reads: selecting settings for maximizing number of reliable loci

Using the settings *m* (minimum number of identical, raw reads required to create a stack) = 10, *M* (number of mismatches allowed between loci when processing a single individual) = 1, and *n* (number of mismatches allowed between loci when building the catalog) = 1 as a starting point, various values were tested for these three parameters (*m* = 3-15, *M* = 1-6 and *n* = 1-6). The setting for max_locus_stacks (maximum number of stacks allowed at a single locus) was 5 to allow four alleles (plus one extra) in each tetraploid individual. The resulting files were loaded into a MySQL database with load_radtags.pl and the amount of reliable loci obtained from each of the runs was compared using the following filters: (1) only allowing loci with 1-10 SNPs and (2) only counting SNPs that appear in at least 80 % of the individuals. For each parameter the value that maximized the amount of loci was chosen.

References

1 Skrede, I., Carlsen, T., Rieseberg, L. H. & Brochmann, C. Microsatellites for three distantly related genera in the Brassicaceae. *Conserv. Genet.* **10**, 643-648 (2009).

2 Ponce, M. R., Robles, P. & Micol, J. L. High-throughput genetic mapping in *Arabidopsis thaliana*. *Mol. Gen. Genet.* **261**, 408-415 (1999).

3 Bell, C. J. & Ecker, J. R. Assignment of 30 microsatellite loci to the linkage map of *Arabidopsis*. *Genomics* **19**, 137-144 (1994).

4 Suwabe, K., Iketani, H., Nunome, T., Kage, T. & Hirai, M. Isolation and characterization of microsatellites in *Brassica* rapa L. *Theor. Appl. Genet.* **104**, 1092-1098 (2002).

5 Clauss, M. J., Cobban, H. & Mitchell-Olds, T. Cross-species microsatellite markers for elucidating population genetic structure in *Arabidopsis* and *Arabis* (Brassicaeae). *Mol. Ecol.* **11**, 591-601 (2002).

6 Uzunova, M. I. & Ecke, W. Abundance, polymorphism and genetic mapping of microsatellites in oilseed rape (*Brassica napus* L.). *Plant Breeding* **118**, 323-326 (1999).

7 Arumuganathan, K. & Earle, E. D. Estimation of nuclear DNA content of plants by flow cytometry. *Plant Mol. Biol. Rep.* **9**, 229-241 (1991).

8 Doležel, J., Greilhuber, J. & Suda, J. Estimation of nuclear DNA content in plants using flow cytometry. *Nature Protoc.* **2**, 2233-2244 (2007).

9 Temsch, E. M., Greilhuber, J. & Krisai, R. Genome size in liverworts. *Preslia* **82**, 63-80 (2010).
